# Use of allogeneic freeze-dried conditioned serum for the prevention of degradation in cartilage exposed to IL-1ß

**DOI:** 10.1186/s12917-022-03227-2

**Published:** 2022-07-11

**Authors:** Livia Camargo Garbin, C. Wayne McIlwraith, David D. Frisbie

**Affiliations:** 1 Orthopaedic Research Center, Colorado State University, 2350 Gillette Drive, Fort Collins, CO 80523 USA; 2 Present affiliation: Department of Large Animal Medicine, College of Veterinary Medicine, University of Georgia, 501 D.W. Brooks Drive, Athens, GA 30602 USA; 3 C. Wayne McIlwraith Translational Medicine Institute, 2350 Drive, Fort Collins, CO 80523 USA

## Abstract

**Background:**

Autologous conditioned serum (ACS) has been extensively used in the field of veterinary orthopaedics and sports medicine. Due to the autologous and blood-derived nature of this product, issues such as individual variability, need for storage at low temperatures and non-availability for immediate are frequently encountered for ACS use in the field. To address those issues, we proposed the evaluation of an off-the-shelf allogeneic freeze-dried version of conditioned serum in an in vitro model of osteoarthritis. In this study, we evaluated if origin (autologous and allogeneic) and preparation (frozen and freeze-dried) of conditioned serum could influence in its effect in an in vitro model.

**Results:**

IL-1β stimulation in cartilage led to a significant increase in media GAG and decreased levels of GAG in cartilage explants at the termination of the experiment. No significant differences were noted in outcomes measured in the cartilage explants with respect to the main effects of treatment (frozen versus freeze-dried serum), autologous versus allogeneic preparations or based on serum concentration.

**Conclusions:**

The study did not observe any substantial differences in the response of cartilage to allogeneic freeze-dried CS when compared to other independent parameters (autologous and frozen preparations). Further investigation using in vivo systems appears warranted.

**Supplementary Information:**

The online version contains supplementary material available at 10.1186/s12917-022-03227-2.

## Background

Inflammation within the joint is a common feature in equine and human osteoarthritis (OA) and it has been associated with its symptoms and as a trigger point for the progressive degeneration of the joint [1]. In traumatically injured joints, acute synovitis and capsulitis may contribute to joint degradation through the release of inflammatory mediators and enzymes, which has been demonstrated firstly in the horse [1]. For this reason, more interest has been given to drugs that could modulate OA development, including the inhibition of target cytokines involved in inflammatory pathways [2]. Although IL-1 is not the only pro-inflammatory protein enrolled in OA [3] pathogeneses, studies suggest its action is the most promising approach for cytokine blocking [4]. IL-l receptor antagonist protein (IL-1Ra) is a naturally occurring molecule that competes for occupancy of the IL-1 cell surface receptors, but doesn’t initiate the catabolic response typical of IL-1 [5]. Thus IL-1Ra is a natural inhibitor of IL-1 and its efficacy has been shown in vivo, including in an equine model [6]. Autologous conditioned serum (ACS) is produced following culture of whole blood in the presence of medical grade coated glass beads. This process results in serum that is enriched in endogenous IL-1Ra as well as anti-inflammatory cytokines (such as IL-4, IL-10) and growth factors [7].

While significant lameness and histological improvement was observed in experimentally induced osteoarthritic joints treated with ACS commercial kits in horses [8] multiple limitations preclude its widespread acceptance and use. One limitation of ACS is the preparation and need for storage at low temperatures(<− 20 °C), which is inconvenient for most equine practitioners. Additionally, ACS presented variation in both pro- and anti-inflammatory cytokine based on the patient [7]. The current work addresses some of these issues. Specifically, this study evaluated allogeneic freeze-dried conditioned serum (CS), allowing a more homogeneous, stable, quantifiable and practical option for use in the field. Thus, the objective of this research was to determine the influence of origin (autologous and allogeneic) and preparation (freeze-dried and frozen) in the ability of conditioned serum (CS) to palliate degradative changes induced by IL-1ß on equine cartilage.

This study is based on three hypotheses tested in a cartilage explant in vitro model. Firstly, that freeze-dried CS would not present significantly different effects compared to frozen preparations. The second is that allogeneic CS would not present significantly different effects compared to autologous preparations and the third, that CS effects would be concentration dependent.

## Results

### Stimulation of cartilage explants with IL-1β (analysis 1)

The efficiency of using IL-1β as a model to induce a pro-inflammatory environment for cartilage was assessed in this analysis. The presence of IL-1β caused a significant increase in glycosaminoglycan (GAG) released in culture supernatants at Days 2, 4 and 6 (*P* < 0.005) independent of the type of media supplementation used (Supplementary Table 1). In cartilage explants, the presence of IL-1β (34.32 ± 9.52 μg/μg of DNA) resulted in significant GAG loss at the end of the study compared to control (69.09 ± 9.84 μg/μg of DNA; *P* = 0.0143).

A significant effect of IL-1β was observed for ^35^SO_4_-labeled GAG in culture supernatant at Day 2 and 4 (*P* < 0.0339) of the experiment (Supplementary Table 2). The interaction between type of media used and effect of L-1β was as well significant at Day 2 and 6 (*P* < 0.034).

### CS effect on cartilage stimulated with IL-1β (analysis 2)

This analysis was done to assess the effect of CS in cartilage under IL-1β stimulation.

**Fig. 1:**
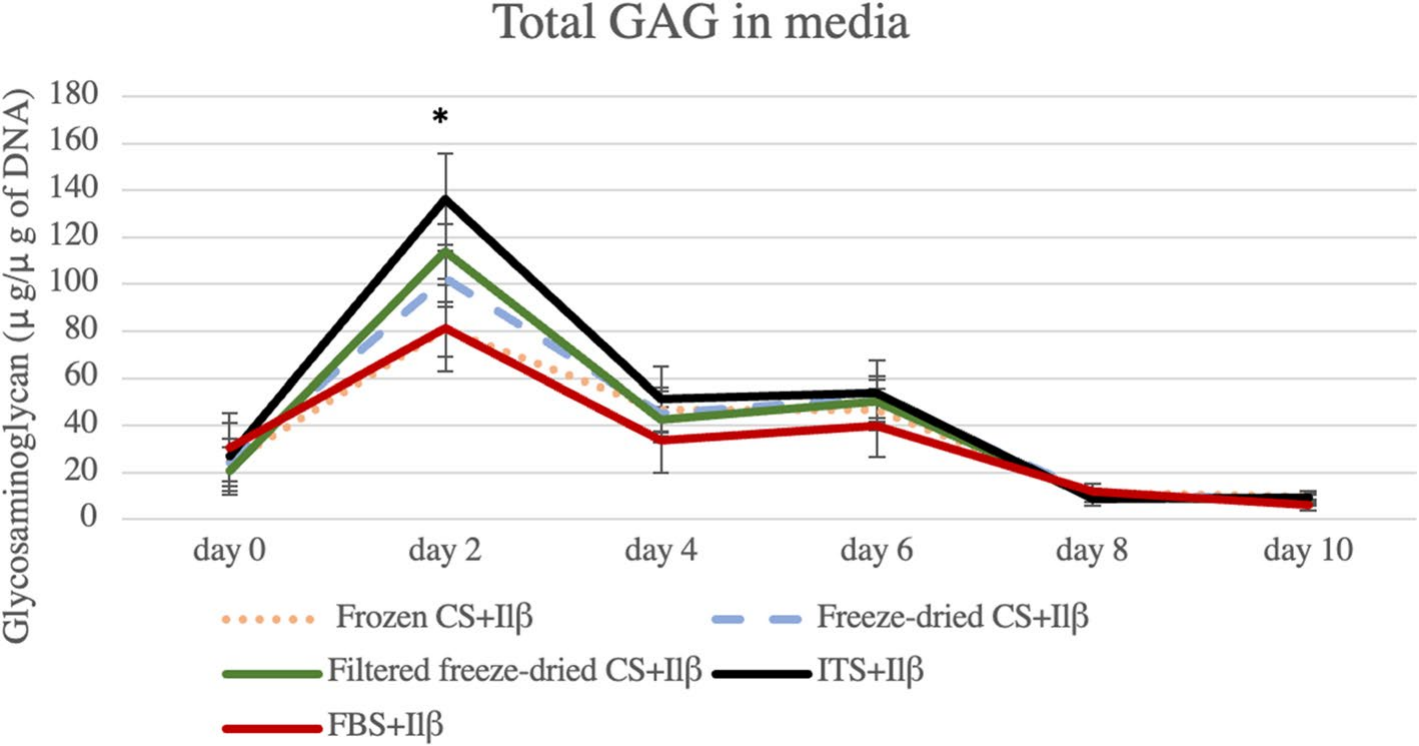
Total Glycosaminoglycan (GAG) in media during the experiment. A significant increase in GAG released in media was observed at Day 2 (*P* = 0.0133). Control samples in ITS media had significantly more GAG released compared to CS treated samples, being only significant when compared to Frozen CS treated samples. Results are represented as mean and bars represent standard error of the mean. Asterisks demonstrate statistical significance between the groups on Day 2. Levels of significant was considered *P* ≤ 0.05

In culture supernatant GAG analysis, a significant effect of Treatment was observed only at Day 2 of the experiment (*P* = 0.0133). Specifically, ITS+IL-1β control presented increase GAG levels in media compared to Frozen CS (Fig. 1). No significant differences between Frozen CS and either Freeze-dried preparation were observed in this comparison. In cartilage explants however, no significant differences were observed in total GAG.

**Fig. 2:**
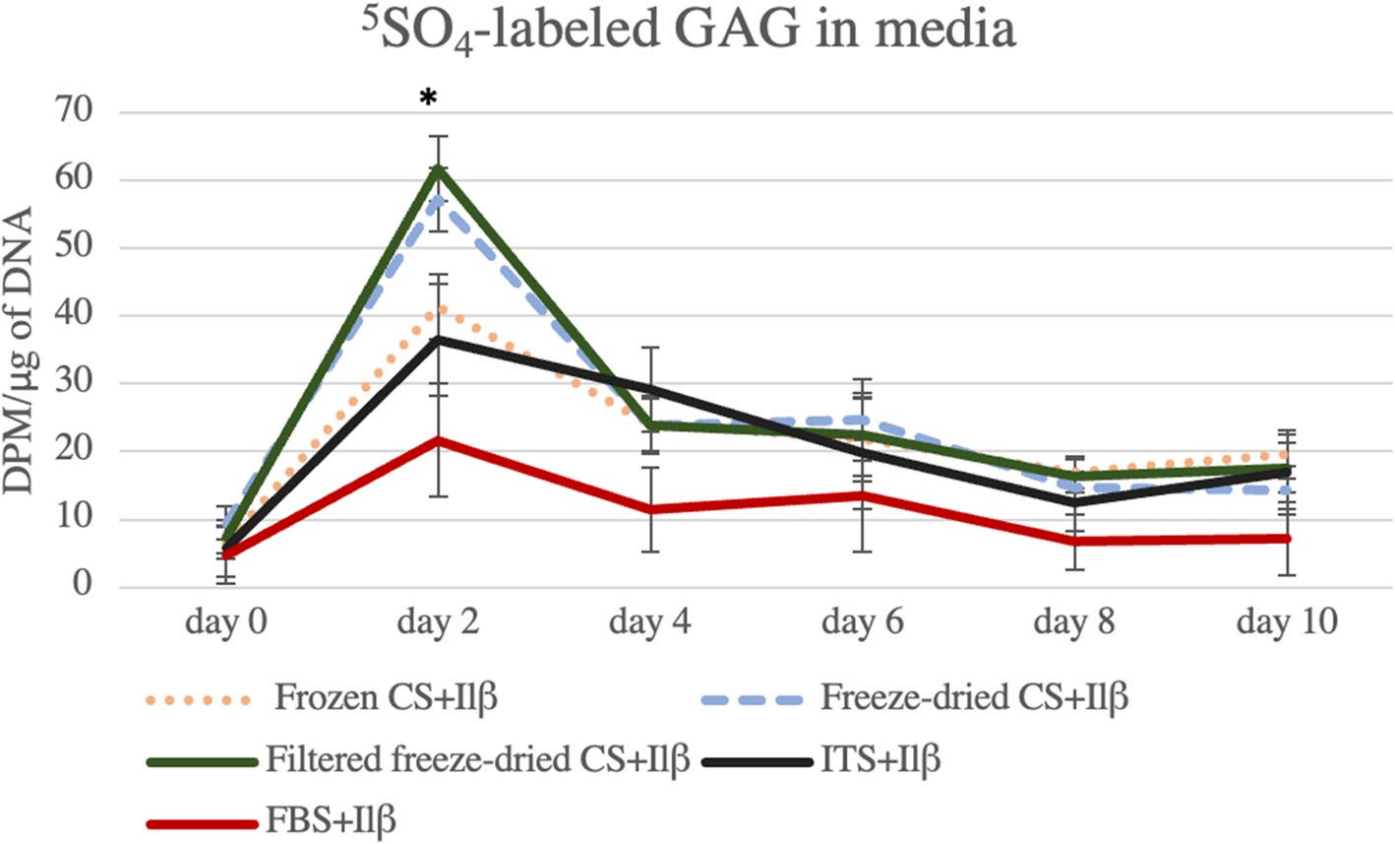
^**3**5^SO_4_-labeled GAG released in media during the experiment**.** ^35^SO_4_-labeled GAG presented significantly higher levels in media only at Day 2 (*P* ≤ 0.0001), in samples treated with Freeze-dried and Filtered Freeze-dried CS in comparison with the ITS+IL-1β and FBS+ IL-1β controls and Frozen CS groups. Results are represented as mean and bars represent standard error of the mean. Asterisks demonstrate statistical significance between the groups on Day 2. Levels of significant was considered *P* ≤ 0.05

The presence of CS had a significant effect (*P* ≤ 0.0001) on ^35^SO_4_-labeled GAG in media only at Day 2. Specifically, increased levels of ^35^SO_4_-labeled GAG in media were observed in samples treated with Freeze-dried (57.25 ± 4.69 DPM/ug of DNA) and Filtered Freeze-dried CS (61.75 ± 4.75 DPM) in comparison with the ITS+IL-1β (36.48 ± 8.34 DPM/μg of DNA), FBS+ IL-1β (21.63 ± 8.34 DPM/μg of DNA) controls and Frozen CS group (41.37 ± 4.69 DPM/μg of DNA) (Fig. 2). These changes did not result in any significant differences in the ^35^SO_4_-labeled GAG in cartilage explants at the termination of the study when compared to ITS+IL-1β control.

### Study of the effects of CS different formulations in cartilage explants

#### Biochemical data analysis (analysis 3)

**Fig. 3:**
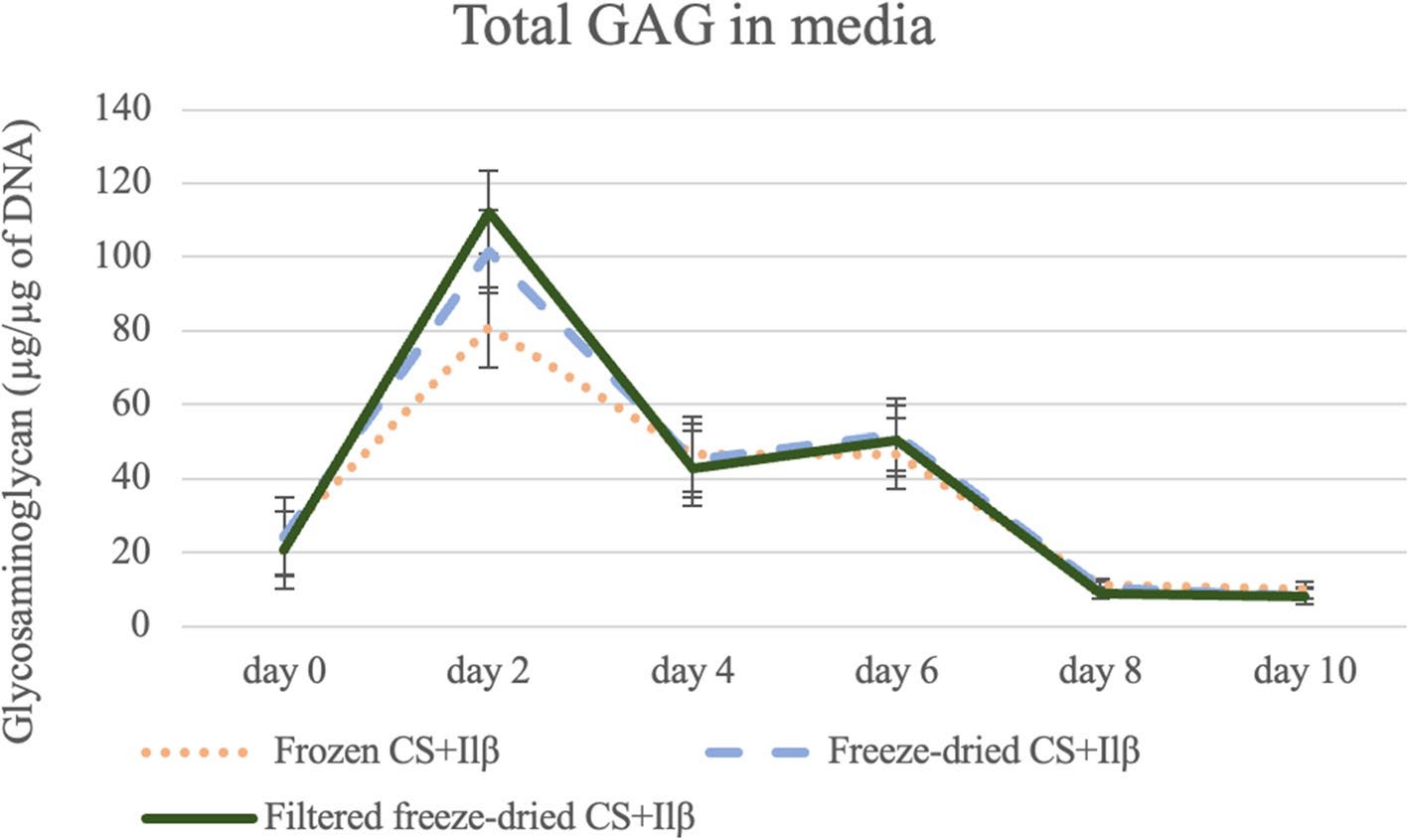
Treatment comparison for the total glycosaminoglycan (GAG) in media during the experiment. Decreased levels of media GAG were observed in the media of Frozen CS compared to either Freeze-dried CS treatments, not being statistically significant. Results are represented as mean and bars represent standard error of the mean. Levels of significant was considered *P* ≤ 0.05

**Fig. 4:**
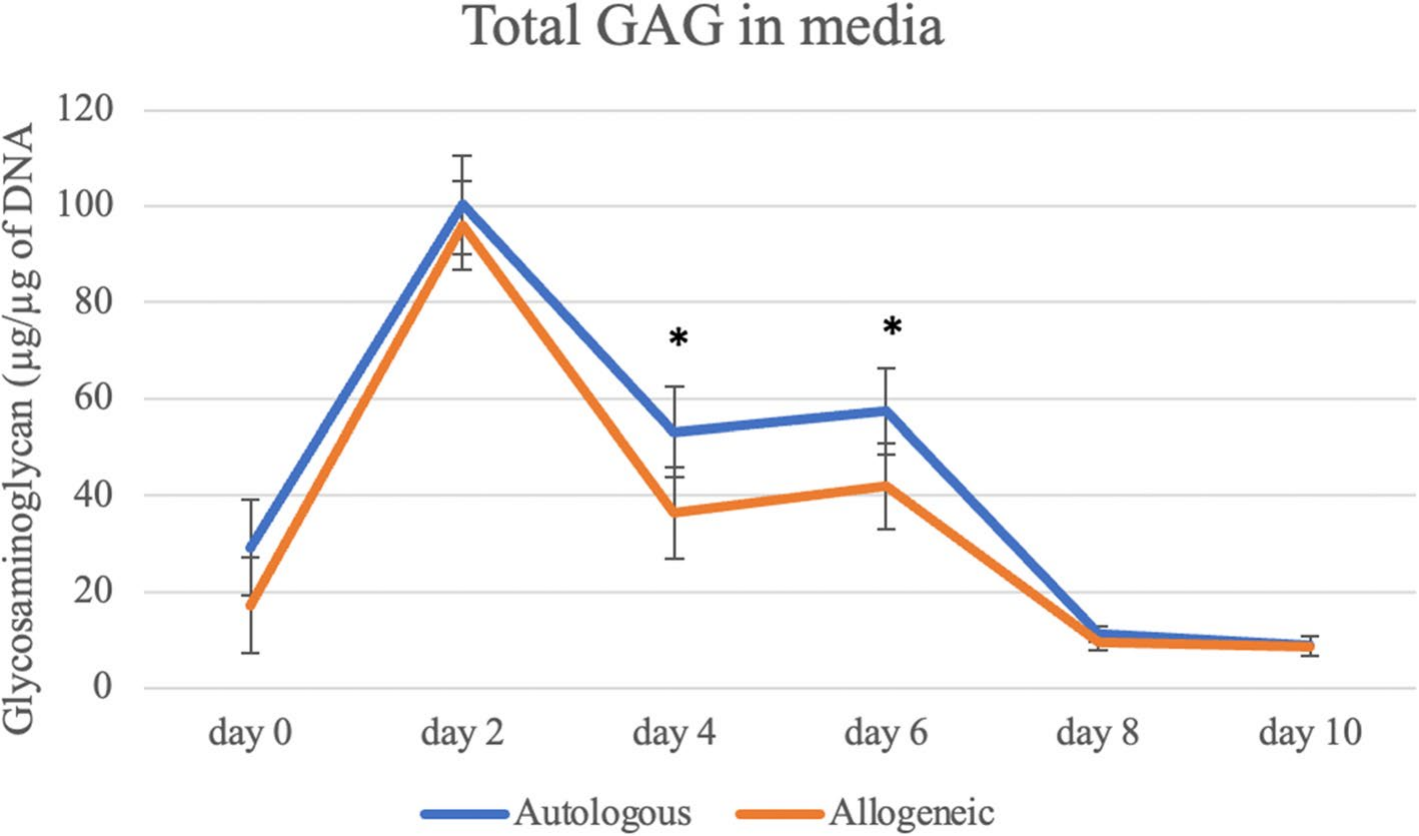
Comparison between autologous and allogeneic groups for the total glycosaminoglycan (GAG) in media during the experiment. On Days 4 and 6 of the experiment, Allogenicity had significant effect (*P* < 0.022). Decreased levels of GAG were observed in Allogeneic treated samples when compared to Autologous treated samples on these days. Results are represented as mean and bars represent standard error of the mean. Asterisks demonstrate statistical significance between the groups on Days 4 and 6. Levels of significance was considered *P* ≤ 0.05

In this analysis, only CS treated samples were considered to assess the main effects Treatment, Allogenicity and Concentration. Similar to the results observed in Analysis 2 lower media GAG was observed in the media of Frozen CS compared to either Freeze-dried CS treatments; however, in this comparison the *P*-value was close to significant (*P* = 0.0512) (Fig. 3). On Days 4 and 6 of the experiment, Allogenicity had significant effect (*P* < 0.022), with less GAG being measured in the media of Allogeneic treated samples (Day 4: 36.38 ± 9.43; Day 6: 41.98 ± 8.98) when compared to Autologous (Day 4: 53.17 ± 9.39; Day 6: 57.38 ± 8.98) on these days (Fig. 4). These changes did not result in any significant differences observed in the total cartilage explants GAG at the end of the experiment.

**Fig. 5:**
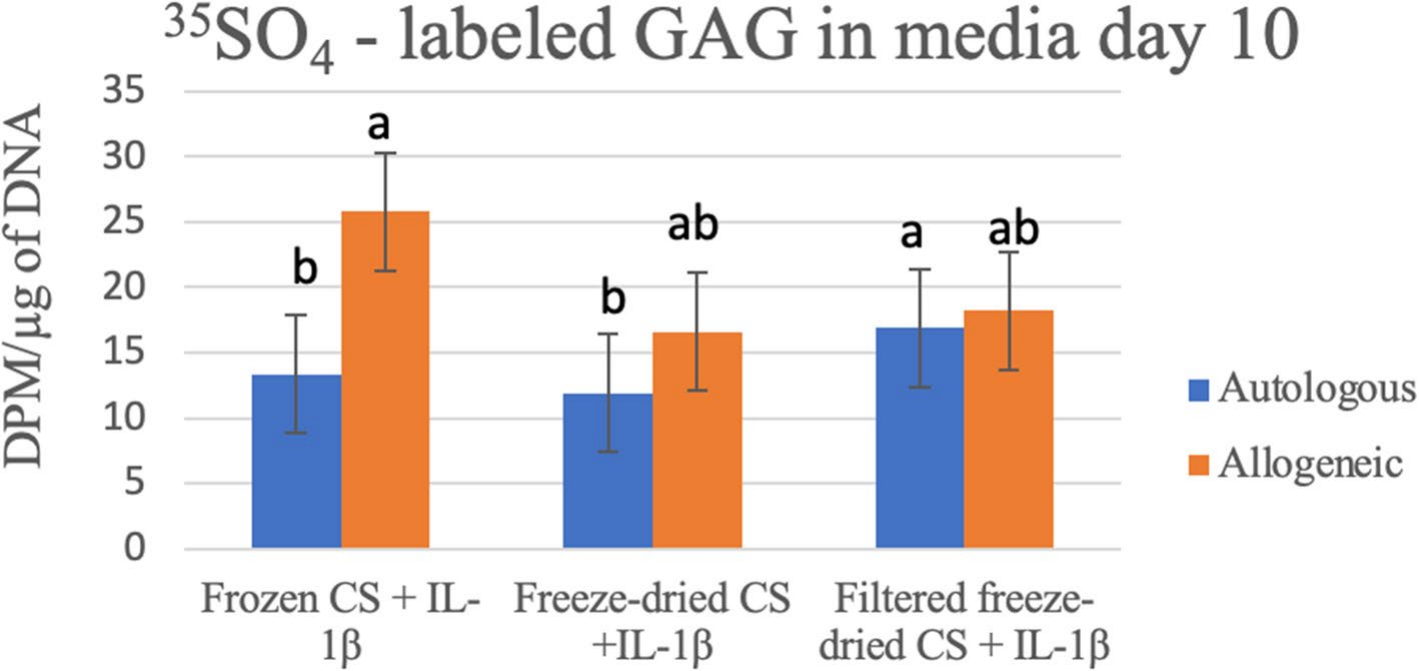
^35^SO_4_ – labelled GAG in media day 10 for Treatment*Allogenicity main effect interaction. Allogeneic Frozen CS treated samples had significantly more ^35^SO_4_ – labelled GAG in media compared to Autologous Frozen and Freeze-dried CS. Results are represented as mean and bars represent standard error of the mean. Bars with different letters indicate a significant difference between groups. Level of significance used 0.05

The amount of ^35^SO_4_-labeled GAG in culture supernatant demonstrated a statistical significant difference on Day 2 based on CS Treatment (*P* = 0.0029) similar to the results presented in Analysis 2. The concentration of ^35^SO_4_-labeled GAG in media on Day 10 demonstrated a significant interaction (*P* ≤ 0.011) for individual comparisons between Allogenicity and Treatment effects. Specifically, it appeared more labelled GAG was released into the media with Allogeneic Frozen CS compared to Autologous Frozen CS as well as Autologous Freeze-dried CS (Fig. 5). However by the termination of the study no significant differences in cartilage ^35^SO_4_- labelled GAG were observed.

#### Gene expression

**Fig. 6:**
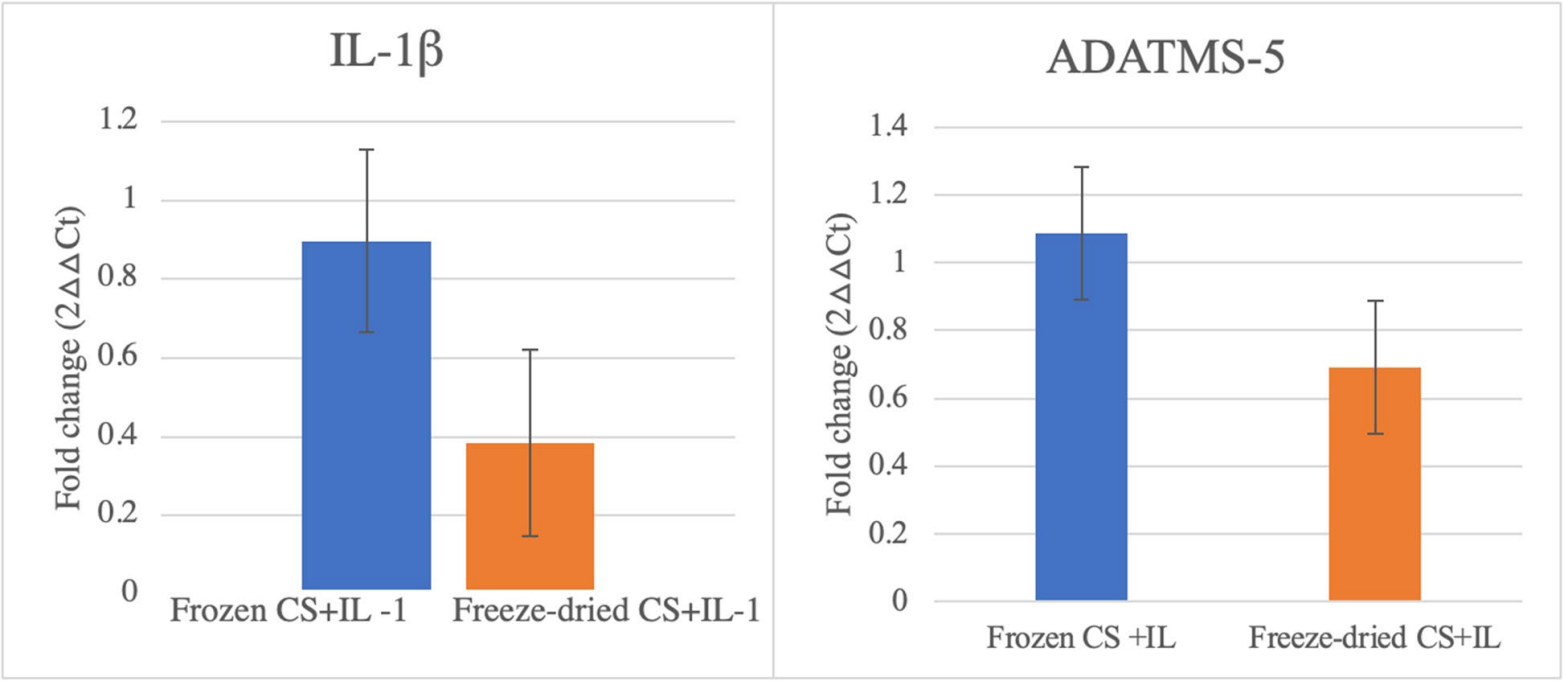
Relative expression of different genes evaluated in this experiment. ADAMTS-5 and IL-1 β were evaluated in cartilage after the termination of the study. No significant difference was observed between samples treated with difference CS groups, for the main effects in this study including Treatment. The relative expression for the genes studied did not present a normal distribution, and data was log transformed to fit the assumption of normality. The raw fold changes related to ITS+IL-1β controls are presented in the graph for ease of interpretation. Results are demonstrated in means and bars represent standard error of the mean. Level of significance used 0.05

Catabolic cytokines and enzymes related to OA were evaluated for gene expression in this study to assess the modulatory effects of CS in cartilage. Gene expression analysis was performed on log transformed values to meet the assumptions of normality. No changes were greater than 2-fold and thus not considered biologically significant for any comparison (Fig. 6).

## Discussion

To the authors’ knowledge, this was the first published study to compare the effects of an allogeneic freeze-dried version of conditioned serum to autologous frozen conditioned serum, in cartilage under inflammatory conditions. This study utilized IL-1β to provide an inflammatory environment. IL-1β resulted in a significant increase in total GAG and ^35^SO_4_-labeled GAG release from cartilage explants, independent of the type of media. When added to media, IL-1β resulted in less total GAG within the explants compared to non-IL-1β stimulated samples as expected, although not to the same extent as previously observed when IL-1β was continually present in the media [9, 10]. In those studies, cartilage explants were continuously stimulated with IL-1β as opposed to the acute exposure to the IL-1β used (Day 0 and 4), which may explain the differences. Although not all outcome parameters showed significant effect of IL-1β stimulation, the data in this experiment suggests a significant catabolic effect of this cytokine, supporting the model in this study.

In the second analysis, when comparing the CS treated groups in cartilage to ITS+IL-1β controls, differences were seen at Day 2 of the study but these changes did not result in differences that affected the cartilage explants at the termination of the study. While little evidence of protective effects of CS in the current in vitro study was observed in cartilage, significant clinical and histological improvement was detected in vivo in joints with OA in previous reports [8, 11]. It has also been reported that ACS combined with physiotherapy demonstrated clinical improvement up to 2 years after treatment in human OA knees [12]. Autologous conditioned serum also improved joint function and reduced shoulder pain more effectively than betamethasone [13]. The model used in the current study lacked the synovium and other in vivo mechanisms that could be key to the ACS effects.

Additionally, it has been reported that IL-1Ra:IL-1β ratio needs to range between 10 to 100-fold to inhibit the bioactivity of IL-1β [14]. Based on these numbers IL-1Ra in the current study may have been below this threshold. While the goal of the current study was not to assess raw concentrations of IL-1Ra, it has been measured in previous experiments and found to be 11.122 to 50.11 ng/mL (data not shown). These levels of IL-1Ra would be below what would be expected to block the IL-1β (10 ng/mL) when one considers the CS was no greater than 30% of the media; thus, providing another possibility for not seeing a robust treatment effect in the current model. It is important to consider that 10 ng/mL of IL-1 β is a supra-physiological concentration beyond of what was found in joints with arthritis (ranging from 25 pg/mL to 175 pg/mL) [15]. Therefore the insufficient protective effects in this in vitro model does not imply in lack of clinical effect in vivo.

For the main effect of Concentration accessed in this experiment, two concentrations of CS were studied (10 and 30%) in media. No significance was found for GAG or ^35^SO_4_-labeled GAG in cartilage or culture supernatant during the experiment, which suggests that the effects of CS in cartilage were not concentration dependent. These findings are in accordance with previous research in which the total GAG present in chondrocyte pellets stimulated with IL-1β was not demonstrated to be significant with the use of different CS concentrations [16].

The study did not show any significant differences in the cartilage explant parameters measured at the termination of the study as it related to the effect of Allogenicity, despite some differences at Day 2 and 10. To the authors’ knowledge no studies comparing the effects of autologous and allogeneic conditioned serum have been published. However, other biological therapies, specifically platelet-rich plasma (PRP), demonstrated similar effects in allogeneic compared to autologous versions in in vivo [17] and in vitro [18].

Similarly no significant effects of lyophilization of CS were observed in cartilage at the termination of the study. Although no studies published at this date focused on the effects of lyophilized conditioned serum as potential modulatory treatment, freeze-drying has been performed in other blood derived products [18]. Freeze-drying did not demonstrate to interfere with granulation, proliferation and angiogenic response of platelet-derived products in a wound mouse model [19].

Based on the findings of this experiment, the presentation of the conditioned serum proposed (allogeneic freeze-dried) demonstrated to be equivalent to the current version used in the field (autologous frozen). This is the first step for the development of a more practical form of using biologics in the field. An off-the-shelf version of conditioned serum would allow the immediate use of blood derived products, without the need of blood collection and handling, processing, incubation period or storage at low temperatures. This would allow the field clinician, who may not have immediate access to laboratory facilities and equipment to use biologic products in a much more convenient matter.

Some potential pitfalls should be noted in the current study. The complexity and sample numbers of the study design did not allow the collection of all possible outcome parameters. Specifically, histopathologic evaluation was not performed in this study, which could be considered as a potential pitfall. However, GAG analysis in culture supernatant during the experiment and in cartilage allowed the authors to investigate the effects of the proposed formulations of CS in cartilage matrix over time. Another potential pitfall in this study was that growth factors and cytokines present within the different formulations of CS or in the culture supernatant were not measured. It is believed by the authors that each patient’s ACS will be unique and the goal of this study was to compare the freeze-drying technique and overall, allogeneic versus autologous. This lead the investigators to forgo the cytokine composition of the different CS products in the current experiment. We do believe a detailed investigation of the cytokine composition of pooled CS is vital for a better understanding of the product’s effect and potential application in the field, and it is warranted in future research.

Furthermore, in regards to the number of horses used in this experiment for the preparation of CS, we are aware that a greater number of horses could potentially generate a more homogenous product compared to the current study. Previous similar research [20] demonstrated that a minimum of 5 horses would be sufficient to provide statistical significance for this in vitro study. Based on this information and on availability we included 8 horses to be part of this experiment, and divided those in two groups of 4. This was performed to facilitate logistics for blood collection, preparation of CS and cartilage harvest. Although a greater number of horses could be considered ideal, this study provided only an initial step for the investigation of allogeneic freeze-dried version of CS which already demonstrated favourable results. Future studies should include a greater number of horses to provide more robust results and potentially more homogenous allogeneic product.

## Conclusion

The effects of an allogeneic freeze-dried version of CS overall did not show significantly different outcomes when compared to autologous frozen CS in an inflammatory environment. Further investigation in in vivo models, as well as the detailed study of the cytokine profile of frozen versus freeze-dried CS are warranted to confirm these results.

## Methods

The aim of this study was to evaluate whether the allogeneic freeze-dried version of conditioned serum would present similar effects compared to autologous frozen version in an in vitro model of osteoarthritis. In this model, we induced degradative changes in equine cartilage explants using IL-1β and evaluated the protective effects of allogeneic freeze-dried conditioned serum compared to autologous version.

### Animals

Blood was collected from each of the eight skeletally mature and healthy horses (2–5 years of age) for this in vitro study. The animals used in this study were only used for tissue collection and belonged to a terminal research not related to the present study. Blood was harvest for ACS preparation and cartilage was collected after euthanasia. All the procedures were approved by the Universities’ Institutional Animal Care and Use Committee (ACUC, protocol number: 14-5477A). All research involving the horses were carried out in compliance with the ARRIVE guidelines.

### ACS preparation

#### Blood processing and incubation

The ACS was prepared by combining whole blood with medical grade glass beads. Briefly, blood was collected from each horse separately and aliquoted into 50 ml conical tubes containing coated beads for ACS preparation. Blood was incubated for 24 h at 37 °C. Subsequently, the tubes were centrifuged for 10 min at 4000 rpm (1716.88 xg) and serum was aliquoted in smaller volumes and stored in a − 80 °C freezer until the experiment was initiated.

To create the Frozen, Freeze-dried and Filtered freeze-dried treatments, the ACS aliquots from each horse were divided into three equal parts: one part remained frozen at − 80 °C until the experiment was initiated (Frozen treatment), the second portion (Freeze-dried) was lyophilized in specific temperature (< − 45 °C) and pressure (< 100 mtorr) for 18 h (Virtis Sentry Lyophilizer) and samples were then returned to − 80 °C until the commencement of the experiment, the third portion (Filtered freeze-dried treatment) was processed similarly to the freeze-dried group; however, the serum was filtered with a 45 μm syringe filter^1^ before lyophilizing. This latter group was designed to mimic the procedure done clinically (where ACS is sterile-filtered before application), and to verify if filtrating serum could have an impact in its effect. In this study, the effect of the different ACS formulations will be collectively referred as Treatment.

**Fig. 7:**
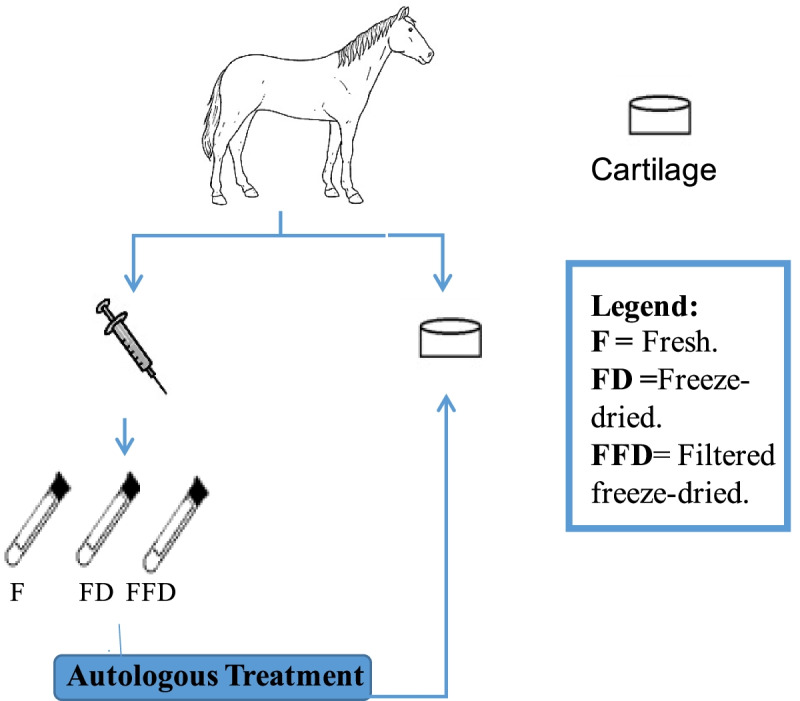
Autologous CS. Blood was collected from eight horses (*N* = 8) and processed to produce the ACS. All the ACS were processed in three different Treatments; Frozen, Freeze- dried and Filtered freeze-dried. The ACS produced was then applied into the cartilage from the same horse the blood was collected. The application of ACS was done in two different concentrations (10 and 30%)

**Fig. 8:**
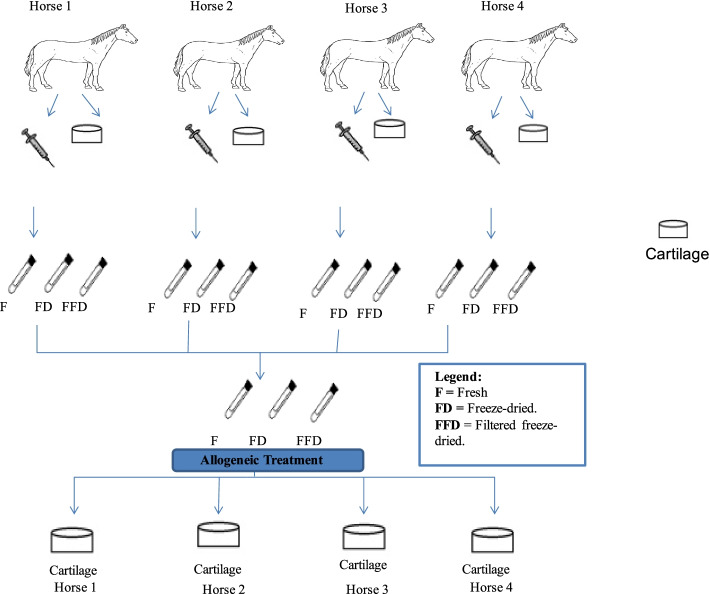
Allogeneic CS. Blood was collected from eight horses (N = 8) and processed to produce the ACS. All the ACS were processed in three different Treatments; Frozen, Freeze- dried and Filtered freeze-dried. Before use, the ACS from each horse of a group of 4 horses (2 groups *N* = 4) was combined before being applied to the explants. The application of the allogeneic CS was done in two different concentrations (10 and 30%)

For the Autologous CS, the ACS produced were kept separated to be applied into the explants of the same horse from which the blood was collected (Fig. 7), while for the Allogeneic conditioned serum (CS), the aliquots from four horses were pooled together (Fig. 8). The effect of Autologous and Allogeneic CS in this study was referred as Allogenicity. The mentioned preparations of CS utilized in this study were used in two different concentrations in this experiment, 10 and 30% in media (volume /volume). The effect of concentration of CS used in this study was referred as Concentration.

### Cartilage explant harvest

Subsequent to euthanasia, cartilage from each horse’s trochlear ridges and condyles of the stifle joint of each horse was harvested using an 8 mm punch and weighted between 70 and 100 mg (wet weight). Cartilage explants were placed in a 24 well plate containing media [DMEM^2^ supplemented with 1 mM nonessential amino acids, 10 mM HEPES, 0.4 mM proline, 0.11 mM ascorbic acid, penicillin (100 U/mL), streptomycin (100 μg/mL) and 200 mM L-alanyl-L-glutamine dipeptide in 0.85% NaCl (GlutaMAX™)^3^]. As cytokines and growth factors in routine FBS media could interfere with the effects of the CS used, a 1% volume/volume of insulin transferrin selenium (ITS; ITS Premix 6.25 mg of insulin, 6.25 mg of transferrin and 6.25 μg of selenium acid, 6.26 mg of transferrin and 6.25 μg of selenium acid)^4^ [21–23] supplement was chosen for this experiment. A FBS media control utilized to allow comparison to other studies. The effects of media supplementation (ITS or FBS) was collectively referred in this study as Media. All explants were allowed to equilibrate in the designated medium for 48 h in humidified incubator at 37 °C before Treatment application.

### Treatment preparation & tissue culture

CS samples representing Frozen, Freeze-dried and Filtered Freeze-dried were thawed and diluted in ITS media based on intended final CS concentration as well as considering autologous or allogeneic conditions when added to the cartilage explants. All permutations were tested in triplicate within each horse. Control explants were exposed to media supplemented with ITS or FBS. The treatment permutations were applied twice throughout the study on day 0 and day 4. IL-1β was also added to the media on these days to expose explants to an inflammatory condition (IL-1β, R&D Systems)^5^, 10 ng/mL [20, 24] diluted in 0.1% bovine serum albumin (BSA)^6^ and PBS]. Media was changed every 48 h and stored at − 80 °C. On the 10th day of the experiment cartilage explants and the media were collected and frozen at − 80 °C until analysis.

### Biochemical analysis

Cartilage explants were lyophilized and papain digested overnight at 60 °C using a crystallized papain suspension [25].

#### Explant DNA quantification

DNA content in cartilage explants was determined using a fluorescent dye based assay (Hoechst 33258)^7^ [25]. Cartilage samples were run in duplicates and read against a standard curve using calf thymus DNA. The DNA content within the samples was used for normalization of the biochemical data in this study.

#### Explant and culture supernatant GAG quantification

Cartilage explants and culture supernatant were analysed for total GAG content using a modified method of dimethyl methylene blue assay [26]. In brief, the digested samples were added to a dye/buffer solution and read against a standard curve using chondroitin sulfate C from shark cartilage. The DMMB dye binds to sulphated glycosaminoglycan, forming complexes that result in change of colour within the sample. The colour was measured using spectrophotometry, and samples were read at 530 nm in the plate reader. The change in colour intensity is correlated to the amount of glycosaminoglycan found in the sample. All samples and standards were run in triplicate in this assay. GAG was normalized to DNA content in cartilage and presented as GAG in μg / μg of DNA).

#### Explant ^35^SO_4_-labeled proteoglycans quantification

Sixteen hours prior to Day 0, 15 μCi of ^35^SO_4_ was added to the culture supernatant of all cartilage explants to label the newly synthetized GAG. At the termination of the experiment, ^35^SO_4_ within the cartilage was quantified with a modified scintillation count method, using Alcian blue dye as detection system [27, 28]. The samples were run in duplicate and compared to a standard curve that contained different concentrations of ^35^SO_4_ [28]. In brief, samples and standard curve were to each well of a Multiscreen 96 well plate, along with assay buffer and Alcian blue dye. The multiscreen plates were attached to an vacuum manifold which draw the solutions through the screen allowing only the radiolabel bound to GAG and Alcian blue to be catch in the screen. After multiple rinsing steps, the screen of each well of the plate was punched out, and the ^35^SO_45−_ labelled GAG retained in the membrane were quantified by liquid scintillation counting [28]. The activity levels of the ^35^SO_4_ bound to the GAG molecules in cartilage were measured in disintegrations per minute (DPM), and normalized to DNA content (DPM/μg of DNA). This was used as a measure of GAG retention and indirect measure of degradation of newly synthetized GAG.

### Quantitative polymerase chain reaction (qPCR)

To verify the potential modulatory effect of CS in cartilage under inflammatory conditions, gene expression of catabolic enzymes and cytokines related to OA were assessed.

#### Cartilage RNA extraction

After experiment termination, cartilage explants were collected, weighed, immediately placed in guanidinium thiocyanate reagent^8^ and stored at − 80 °C. To perform the RNA extraction, samples were pulverized, homogenized with guanidinium thiocyanate and incubated at room temperature. Nucleic acid was extracted after 20% of chloroform (volume/volume) was added to the samples and followed by centrifugation, according to the manufacturer^8^. Nucleic acids were then precipitated with isopropanol and resuspended in 30 μL of nuclease-free water. All centrifugations were performed at 4 °C.

#### Reverse-transcription

After extraction, RNA was treated for genomic DNA contamination and reverse-transcribed simultaneously using a blend of oligodT and random primers according to manufacturer’s recommendations^9^ and cDNA was stored at − 80 °C until use.

#### qPCR

cDNA solution and Syber®Green^10^ were mixed with each individual primer for the genes of interest and gene expression levels were determined by quantitative real-time PCR. Conditions were: initial activation step 3 min at 95 °C, denaturation 10 s at 95 °C, and annealing 30 s at 60 °C and melting temperature 65 °C–95 °C, incrementing 0.5 °C for 0.5 s. Expression levels of IL-1ß, a desintegrin metalloproteinase with thrombospondin motifs ADAMTS-5 and IL-1ß and ubiquitin C (UBC). All primers were designed using PrimerQuest Tool^11^ and National Center of Biological Information (NCBI)^12^ software. Characteristics used for primer designed were; size under 150 nucleotides, primer should span in an exon-exon junction, having CG% approximately in 50%, melting temperature close to 60 °C and the minimum of hairpins or secondary structures. Efficiencies curves were performed using 10-fold dilutions of cDNA from equine chondrocytes exposed to IL-1β. Primer sequences are shown in Table 1.

**Table 1:** Sequences of primers used in the study

Primers used in the study	Primer sequence (5′ to 3′)
ADAMTS-5	Forward: AAGGTGACTGATGGGACCGAATGT
	Reverse: TTTGAGCCAATGATGCCGTCA
IL-1β	Forward: CCAGAGGCGGCCGGGACATAAC
	Reverse: GGGAAGGCAGCTGGGCATTGATT
UBC	Forward: GGCTGTTAGCTTTTCAGTCTTGTC
	Reverse: CTTAAATTGGGGCTAATGGCTGG

Gene expression levels of the target genes were normalized to the ubiquitin C (UBC) as endogenous control (Δ_CT_). The Δ_CT_ of the target samples were then subtracted from the Δ_CT_ of the calibrator [control samples exposed to IL-1β (ITS+IL-1 β)] resulting in the ΔΔ_CT._ The gene expression was presented in fold (2^-ΔΔCT^) change relative to the calibrator. Samples with a Ct above 38.5 were not considered for analysis.

### Data analysis

A mixed-model analysis of variance was performed in this study using SAS 9.3 software^13^ with three different analyses being performed. First, we evaluated main effect of IL-1β and media supplementation (Media) on explants to test the efficiency of the model. Then, we evaluated the main effect of CS compared to ITS+IL-1 β controls. In this analysis, the effect of CS was averaged over Allogenicity and Concentration. Finally, we compared the effects of Treatment, Allogenicity and Concentration as well as all interaction between these effects in samples exposed to IL-1β. This analysis was performed to evaluate the effect of different CS formulations in the model used.

Culture supernatant GAG and explant GAG and ^35^SO_4_-labeled GAG were considered as dependent variables for all three analyses, and horse was considered as random effect. Outliers were identified based in diagnostic residual plots. Values greater than ±3 standard deviation from the mean were excluded from the data set and statistical analysis was performed again. When sample did not fit assumptions of normality, log transformation was performed and analysis was run again. Restricted Maximum Likelihood was used as estimation technique in this experiment. Individual comparisons supported by the F-test were done using least-squares means procedure. In all statistical comparisons, *P* value ≤0.05 was considered significant.

## Supplementary Information

**Additional file 1: Supplementary Table 1.** Glycosaminoglycan (GAG) in culture supernatant for control samples. **Supplementary Table 2.** Newly synthetized radiolabelled glycosaminoglycan (GAG) released in culture supernatant for control samples.

## Data Availability

The datasets used and/or analysed during current study are available from the corresponding author on reasonable request.

## Abbreviations

ADAMTS-5: A disintegrin and metalloproteinase with thrombospondin motifs 5
FBS: Fetal bovine serum
GAG: Glycosaminoglycan
IL-1β: Interleukin-1β
ITS: Insulin transferrin selenium
OA: Osteoarthritis
PBS: Phosphate Buffered Saline
CS: Conditioned serum
MMP-1: Metalloproteinase-1
DMEM: Dulbecco’s modified Eagle’s medium
μCi: Micro Curies

## Footnotes

1 33mm syringe low binding filter with PES membrane (45 μm), Millipore Sigma, Burlington -MA.

2 DMEM GIBCO, Thermo Fisher Scientific, Waltham, MA.

3 GlutaMAX™Gibco®.

4 ITS Premix 6.25 mg of insulin, 6.25 mg of transferrin and 6.25 μg of selenium acid, 6.26 mg of transferrin and 6.25 μg of selenium acid – BD Bioscience, San Diego- CA.

5 IL-1β, R&D Systems, Minneapolis-MN, USA.

6 BSA, Sigma Chemicals, Saint Louis-MO, USA.

7 Sigma Chemicals, Saint Louis-MO, U.S.A.

8 TRIzol™ Reagent Invitrogen Life Technologies Corporation, Carlsbad-CA, USA.

9 iSript™gDNA Clear cDNA synthesis kit, Bio-Rad, Hercules-CA, USA.

10 iTap™Universal Syber®Green Supermix, Bio-Rad, Hercules-CA, USA.

11 PrimerQuest Tool. IDT-Integrated DNA Technologies.

12 NCBI- National Center of Biotechnology Information.

13 *SAS/STAT 9.3 user’s guide*. Carey, NC: SAS Institute Inc.,2011;515p.

## References

[1] McIlwraith CW, McIlwraith CW, Frisbie DD, Kawcak CE, van Weeran PR (2016). Traumatic arthritis and posttraumatic osteoarthritis in the horse. Joint disease in the horse.

[2] Frizziero AGE, Giannotti OF, Masiero S, Maffulli N. Autologous conditioned serum for the treatment of osteoarthritis and other possible applications in musculoskeletal disorders. Br Med Bull. 2013. 10.1093/bmb/lds016. 10.1093/bmb/lds016 22763153

[3] Hayami T (2008). Osteoarthritis of the knee joint as a cause of musculoskeletal disability symptom complex(MADS). Clin Calcium.

[4] Calich AL, Domiciano DS, Fuller R. Osteoarthritis: can anti-cytokine therapy play a role in treatment? Clin Rheumatol. 2010. 10.1007/s10067-009-1352-3. 10.1007/s10067-009-1352-3 20108016

[5] Dinarello CA, Thompson RC. Blocking IL-1: interleukin-1 receptor antagonist in vivo and in vitro. Immunol Today. 1991. 10.1016/0167-5699(91)90142-G. 10.1016/0167-5699(91)90142-G 1838480

[6] Frisbie DD, Ghivizzani S, Robbins PD, et al. Treatment of experimental equine ostearthritis by in vivo delivery of the equine interleukin-1 receptor antagonist gene. Gene Ther. 2002. 10.1038/sj.gt.3301608. 10.1038/sj.gt.3301608 11850718

[7] Hraha TH, Doremus KM, McIlwraith CW, et al. Autologous conditioned serum: the comparative cytokine profiles of two commercial methods (IRAP and IRAP II) using equine blood. Equine Vet J. 2011. 10.1111/j.2042-3306.3010.00321.x. 10.1111/j.2042-3306.2010.00321.x 21496084

[8] Frisbie DD, Kawcak CE, Werpy NM, et al. Clinical, biochemical, and histologic effects of intra-articular administration of autologous conditioned serum in horses with experimentally induced osteoarthritis. Am J Vet Res. 2007. 10.2460/ajvr.68.3.290. 10.2460/ajvr.68.3.290 17331019

[9] Takafuji VA, McIlwraith CW, Grodzinsky AJ, Frisbie DD. Effects of interleukin-1 alpha ad interleukin-1 beta on proteoglycan metabolism and prostaglandin E2 synthesis in equine cartilage explants. AJVR. 2002. 10.2460/ajvr.2002.63.551. 10.2460/ajvr.2002.63.551 11939318

[10] Frisbie DD, Nixon AJ (1997). Insulin-like growth factor 1 and corticosteroid modulation of chondrocyte metabolic and mitogenic activities in interleukin 1-conditioned equine cartilage. Am J Vet Res.

[11] Baltzer AW, Moser C, Jansen SA, et al. Autologous conditioned serum (Orthokine) is an effective treatment for knee osteoarthritis. Osteoarthr Cartil. 2009. 10.1016/j.joca.2008.06.014. 10.1016/j.joca.2008.06.014 18674932

[12] Baselga Garcia-Escudero J, Trillos MH, P. Treatment of osteoarthritis of the knee with a combination of autologous conditioned serum and physiotherapy: a two-year observational study. PLoS One. 2015. 10.1371/journal.pone.0145551. 10.1371/journal.pone.0145551 PMC4692499 26709697

[13] Damjanov N, Barac B, Colic J, et al. The efficacy and safety of autologous conditioned serum (ACS) injections compared with betamethasone and placebo injections in the treatment of chronic shoulder joint pain due to supraspinatus tendinopathy: a prospective, randomized, double-blind, controlled study. Med Ultrason. 2018. 10.11152/mu-1495. 10.11152/mu-1495 30167587

[14] Firestein GS, Boyle DL, Yu C, et al. Synovial interleukin-1 receptor antagonist and interleukin-1 balance in rheumatoid arthritis. Arthritis Rheum. 1994. 10.1002/art.1780370507. 10.1002/art.1780370507 8185691

[15] Vangsness CT, Burke WS, MacPhee RD (2006). Synovial fluid analysis.

[16] Carlson ER, Stewart AA, Carlson KL, et al. Effects of serum and autologous conditioned serum on equine articular chondrocytes treated with interleukin-1β. Am J Vet Res. 2013. 10.2460/ajvr.74.5.700. 10.2460/ajvr.74.5.700 23627382

[17] Zhang ZY, Huang AW, Fan JJ, et al. The potential use of allogeneic platelet-rich plasma for large bone defect treatment: immunogenicity and defect healing efficacy. Cell Transplant. 2013. 10.3727/096368912X653183. 10.3727/096368912X653183 22863146

[18] Camargo Garbin L, McIlwraith CW, Frisbie DD. Evaluation of allogeneic freeze-dried platelet lysate in cartilage exposed to interleukin 1-β in vitro. BMC Vet Res. 2019. 10.1186/s12917-019-2118-z. 10.1186/s12917-019-2118-z PMC6824121 31675958

[19] Pietramaggiori G, Kaipainen A, Czeczuga JM, Wagner CT, Orgill DP (2006). Freeze-dried platelet-rich plasma shows beneficial healing propertie in chronic wounds. Wound Repair Regen.

[20] Kisiday JD, McIlwraith CW, Rodkey WG, et al. Effects of platelet-rich plasma composition on anabolic and catabolic activities in equine cartilage and meniscal explants. Cartilage. 2012. 10.1177/1947603511433181. 10.1177/1947603511433181 PMC4297115 26069637

[21] Kisiday JD, Kurz B, DiMicco MA, et al. Evaluation of medium supplemented with insulin-transferrin-selenium for culture of primary bovine calf chondrocytes in three-dimensional hydrogel scaffolds. Tissue Eng. 2005. 10.1089/ten.2005.11.141. 10.1089/ten.2005.11.141 15738669

[22] Kawcak CE, Trotter GW, Frisbie DD (1996). Maintenance of equine articular cartilage explants in serum-free and serum-supplemented media, compared with that in a commercial supplemented medium. Am J Vet Res.

[23] Chua KH, Aminuddin BS, Fuzina NH, et al. Insulin-transferrin-selenium prevent human chondrocyte dedifferentiation and promote the formation of high quality tissue engineered human hyaline cartilage. Eur Cell Mater. 2005. 10.22203/ecm.v009a08. 10.22203/ecm.v009a08 15962238

[24] Takafuji VA, McIlwraith CW, Howard RD. Effects of equine recombinant interleukin-1alpha and interleukin-1beta on proteoglycan metabolism and prostaglandin E2 synthesis in equine articular cartilage explants. Am J Vet Res. 2002. 10.2460/ajvr.2002.63.551. 10.2460/ajvr.2002.63.551 11939318

[25] Kim YJ, Sah RL, Doong JY, et al. Fluorometric assay of DNA in cartilage explants using Hoechst 33258. Anal Biochem. 1988. 10.1016/0003-2697(88)90532-5. 10.1016/0003-2697(88)90532-5 2464289

[26] Farndale RW, Buttle DJ, Barrett AJ. Improved quantitation and discrimination of sulphated glycosaminoglycans by use of dimethylmethylene blue. Biochim Biophys Acta. 1986. 10.1016/0304-4165(86)90306-5. 10.1016/0304-4165(86)90306-5 3091074

[27] Frisbie DD, Kawcak CE, Trotter GW, et al. The assessment of chondrocyte proteoglycan metabolism using molecular sieve column chromatography as compared to three commonly utilized techniques. Osteoarthr Cartil. 1998. 10.1053/joca.1997.0103. 10.1053/joca.1997.0103 9692068

[28] Masuda K., Shirota H., Thonar E.J-M.A. Quantification of ^35^S-labeled proteoglycans complexed to Alcian blue by rapid filtration in multiwell plates. Analytical biochemistry. 1994; 10.1006/abio.1994.1105. 10.1006/abio.1994.1105 7515600

