# Supplementary material for: Use of allogeneic freeze-dried conditioned serum for the prevention of degradation in cartilage exposed to IL-1ß
Source: BMC Vet Res. 2022 Jul 11;18:265. doi: 10.1186/s12917-022-03227-2 (PMC9275241; doi:10.1186/s12917-022-03227-2)
Supplement: Supplementary file 1 — Additional file 1: Supplementary Table 1. Glycosaminoglycan (GAG) in culture supernatant for control samples. Supplementary Table 2. Newly synthetized radiolabelled glycosaminoglycan (GAG) released in culture supernatant for control samples. [file 12917_2022_3227_MOESM1_ESM.docx]

Supplementary Table 1: Glycosaminoglycan (GAG) in culture supernatant for control samples. Cartilage explants were exposed or not to IL-1 β at days 0 and 4 of the experiment. Culture supernatant samples were collected and GAG (µg/µg of DNA) released was evaluated. Different letters denote statistical difference between groups within the same day. Level of significance P$\leq$0.05.

| Glycosaminoglycan in culture supernatant for control samples | | | | | | | |
| --- | --- | --- | --- | --- | --- | --- | --- |
|  | Day 0 | Day 2 | Day 4 | Day 6 | Day 8 | Day 10 | |
| ITS | 5.32±  7.29^b^ | 22.72±  15. 42^c^ | 21.65± 8.83^b^ | 12.31± 8.75^b^ | 7.89± 3.60^a^ | 11.76±  2.63^a^ | |
| ITS+IL-1β | 26.77±  7.29^ab^ | 136.33±  16.42^a^ | 51.13± 8.83^a^ | 54.05± 8.75^a^ | 8.92± 3.60^a^ | 9.08 ±  2.63^ab^ | |
| FBS | 12.02±  7.29^ab^ | 21.06±  15. 42^c^ | 11.062± 9.41^b^ | 12.30± 10.11^b^ | 12.1± 3.60^a^ | 7.53±  2.63^ab^ |  |
| FBS+IL-1β | 30.64±  7.29^a^ | 81.30±  15. 42^b^ | 33.89± 8.83^ab^ | 40.11± 8.75^ab^ | 6.3± 3.60^a^ | 6.20±  2.63^b^ | |

Supplementary Table 2: Newly synthetized radiolabelled glycosaminoglycan (GAG) released in culture supernatant for control samples. Cartilage explants were exposed or not to IL-1 β at days 0 and 4 of the experiment. Culture supernatant samples were collected and radiolabeled GAG (DPM/µg of DNA) released was evaluated. Different letters denote statistical difference between groups within the same day. Level of significance P$\leq$0.05.

| DPM in culture supernatant for control samples | | | | | | |
| --- | --- | --- | --- | --- | --- | --- |
|  | Day 0 | Day 2 | Day 4 | Day 6 | Day 8 | Day 10 |
| ITS | 4.93± 1.28^a^ | 11.35± 4.72^c^ | 12.02± 5.56^b^ | 10.68± 5.67^a^ | 9.35± 2.64^ab^ | 22.30 ±  5.07^a^ |
| ITS+IL-1β | 5.76± 1.28^a^ | 36.48± 4.72^a^ | 29.06 ± 5.56^a^ | 19.82± 5.67^a^ | 12.44± 2.64^a^ | 17.01±  5.07^ab^ |
| FBS | 4.33 ± 1.28^a^ | 26.34 ± 4.72^ab^ | 9.22± 5.56^b^ | 19.42± 5.67^a^ | 4.04± 2.64^b^ | 10.14±  5.07^ab^ |
| FBS+IL-1β | 4.75± 1.28^a^ | 21.63 ± 4.72^bc^ | 11.41±  5.56^b^ | 13.51±  5.67^a^ | 6.66±  2.64^ab^ | 7.17±  5.07^b^ |
